# Microbiota-Gut-Brain Axis Dysregulation in Alzheimer's Disease: Multi-Pathway Effects and Therapeutic Potential

**DOI:** 10.14336/AD.2023.0823-2

**Published:** 2024-05-07

**Authors:** Linkai Qu, Yanwei Li, Fan Liu, Yimeng Fang, Jiaxuan He, Jiahui Ma, Ting Xu, Lei Wang, Pengyu Lei, Hao Dong, Libo Jin, Qinsi Yang, Wei Wu, Da Sun

**Affiliations:** ^1^Institute of Life Sciences & Biomedical Collaborative Innovation Center of Zhejiang Province, Wenzhou University, Wenzhou 325000, China.; ^2^College of Veterinary Medicine, Jilin University, Changchun 130118, China.; ^3^Core Facilities, Zhejiang University School of Medicine, Hangzhou 310058, China.; ^4^College of Life Sciences, Jilin Agricultural University, Changchun 130118, China.; ^5^Wenzhou Institute, University of Chinese Academy of Sciences, Wenzhou 325000, China.; ^6^Key Laboratory for Biorheological Science and Technology of Ministry of Education, State and Local Joint Engineering Laboratory for Vascular Implants, Bioengineering College of Chongqing University, Chongqing 400030, China

**Keywords:** Alzheimer's disease, gut microbiota, nervous system, immune system, neurotransmitter

## Abstract

An essential regulator of neurodegenerative conditions like Alzheimer's disease (AD) is the gut microbiota. Alterations in intestinal permeability brought on by gut microbiota dysregulation encourage neuroinflammation, central immune dysregulation, and peripheral immunological dysregulation in AD, as well as hasten aberrant protein aggregation and neuronal death in the brain. However, it is unclear how the gut microbiota transmits information to the brain and how it influences brain cognition and function. In this review, we summarized the multiple pathways involved in the gut microbiome in AD and provided detailed treatment strategies based on the gut microbiome. Based on these observations, this review also discusses the problems, challenges, and strategies to address current therapeutic strategies.

## 1. Introduction

Alzheimer's disease (AD), a prevalent and progressive neurodegenerative disorder of the central nervous system (CNS), significantly impacts the psychological and social functioning of patients, leading to a decline in their overall quality of life. It is the most common form of dementia observed in older individuals [1]. AD patients frequently exhibit a gradual loss of brain cells and cognitive decline, accompanied by the abnormal accumulation of amyloid β (Aβ) plaques around or outside neurons, as well as the buildup of microtubule-associated protein Tau in cortical neuronal dendrites and axons [2-4]. The accumulation and aberrant aggregation of Tau proteins in neurons lead to reduced microtubule stability, synaptic failure, and disturbance of Ca^2+^ homeostasis, ultimately resulting in apoptosis in neuronal cells [5, 6]. Currently, there are approximately 57.4 million individuals worldwide affected by dementia, and this number is projected to exceed 152.8 million by the year 2050 [7]. Given the escalating prevalence of AD and the substantial treatment burden it imposes, the analysis of AD pathogenesis and the pursuit of effective treatment have emerged as paramount imperatives for researchers. Despite extensive investigations into the etiology of AD, the underlying mechanisms remain incompletely elucidated. Numerous risk factors, including genetics, cerebrovascular disease, hypertension, type 2 diabetes, obesity, dyslipidemia, physical activity, and diet, have been identified, all of which converge on a pivotal element: the gut microbiota [8].

**Figure 1.**
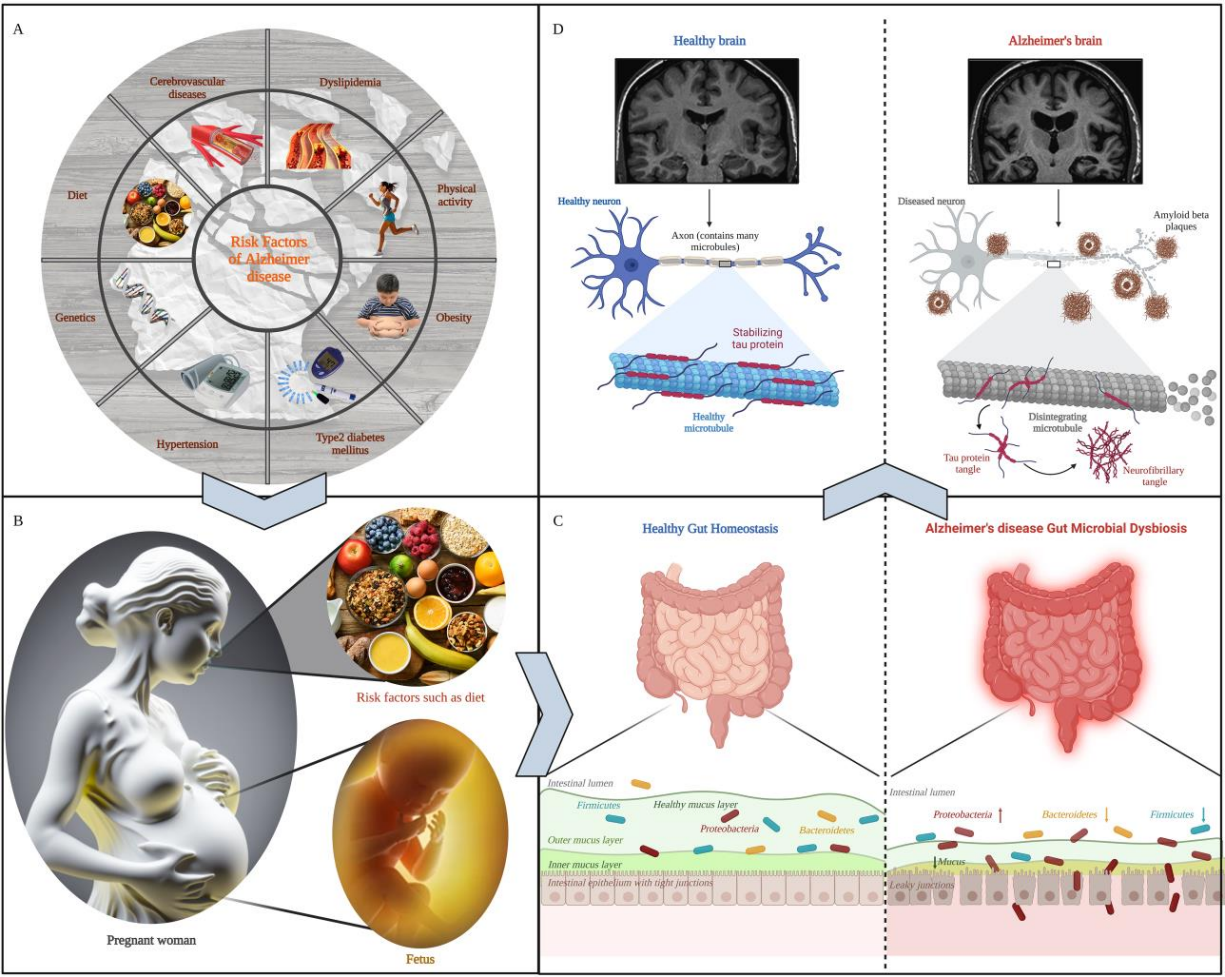
**Multiple risk factors lead to AD by regulating gut microbiota**. (**A**) Multiple risk factors for AD. AD has many risk factors, including heredity, diet and obesity, which greatly increase the incidence and pathological progress of AD. (**B**) The formation of gut microbiota. The fetal gut microbiota is influenced by the maternal microbiota during its initial formation and is differentially influenced by the postnatal environment, breast milk and maternal infections, obesity and other factors on the fetal microbiota. (**C**) Gut composition in healthy and Alzheimer's patients. In patients with AD, the abundance of intestinal beneficial bacteria decreased, the abundance of harmful bacteria increased, the thickness of mucosal layer decreased, and intestinal permeability increased, which led to the entry of pathogenic bacteria and harmful metabolites into the body through intestinal cells and induced inflammation. (**D**) Brain characteristics of healthy and AD patients. The imbalance of gut microbiota accelerated the pathological development of AD, promoted the dissociation of microtubule-associated proteins, the deposition of Aβ protein and neuronal apoptosis.

The presence of an imbalance in intestinal microflora, a reduction in the abundance and number of beneficial bacteria, is frequently observed in patients with AD. This observation suggests that the intestinal flora plays an indispensable role in the pathological progression of AD [9]. This condition gives rise to enduring inflammation and intestinal metabolic issues, alongside the deterioration of the intestinal barrier. Concurrently, bacteria and their byproducts exploit this opportunity to exacerbate insulin resistance (IR), glucose metabolism dysfunction, and neuronal demise, thereby inflicting damage upon the brain and other organs, ultimately fostering the onset of AD and other disorders [10]. Consequently, the microbiota gut-brain axis (MGBA) has been posited as a prospective target for the treatment of neurodegenerative disorders such as AD and has emerged as an innovative therapeutic approach [11].

The establishment of the microbial community within the human body commences during birth, wherein the fetus is exposed to the maternal gut microbiota while in utero [12, 13]. Research has demonstrated that the mode of delivery, whether natural birth or cesarean section, exerts an influence on the microbial community of neonates, leading to notable disparities in the composition of the microbiome [14, 15]. Furthermore, apart from the mode of delivery, a multitude of factors during the initial phases of life, such as prematurity, feeding regimen, host genetics, environmental conditions, maternal inheritance, infections, obesity, stress, and exposures to both antibiotics and non-antibiotic substances, exert diverse levels of impact on the microbiota composition of neonates [12]. Therefore, the microbiota is distinct and uniform for each individual, primarily localized in the gastrointestinal tract, and plays a crucial role in preserving host homeostasis and overall well-being. The intricate communication pathways linking the gastrointestinal tract and the brain involve the CNS, the enteric nervous system (ENS), and the intestinal microflora, collectively known as the MGBA [16]. The gut microbiota communicates bidirectionally with the brain through the neural, humoral, immune, and endocrine systems and influences the brain's physiology, cognition and behavior (Fig. 1).

This paper aims to examine the multi-pathway role of intestinal flora in AD, encompassing its impact on both the immune and nervous systems, as well as the intricate small molecular metabolic system that exists between microorganisms and the brain. Additionally, this study systematically summarizes the various approaches to directly or indirectly intervene in gut flora, thereby potentially influencing the development and progression of AD. Furthermore, this paper thoroughly examines the complexities associated with interventions targeting gut flora in the context of AD. It also puts forth potential remedies, highlighting the significance of investigating the role and therapeutic efficacy of gut flora in AD research. This study anticipates that further exploration in this area will yield substantial evidence to enhance and address the treatment of AD.

## 2. Multi-pathway effect of intestinal flora on AD

There are multiple pathways, encompass intricate neural interactions and meticulously regulated neuronal pathways, as well as a subtle and challenging to detect small molecular information transmission system, that can facilitate the transfer of information and interactions between the brain and the gut microbiota. This relationship has emerged as a highly relevant factor in the context of AD.

### 2.1 Nervous system pathway

**Figure 2.**
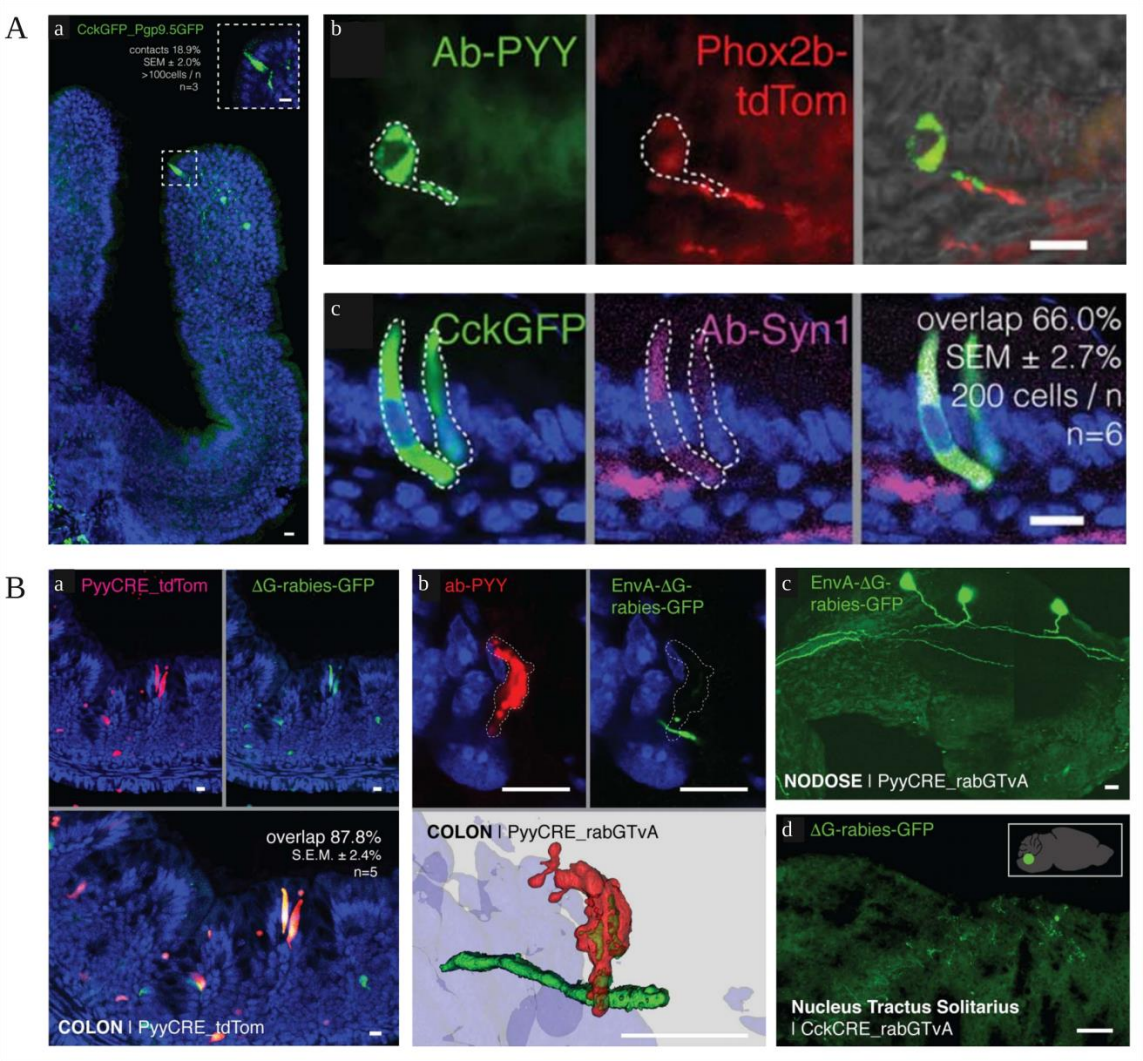
**Enterocerebral neural pathways of the intestine and brain**. (**A**) Enteroendocrine cells contact sensory nerve fibers. (**B**) Enteroendocrine cells of the colon and small intestine synapse interact with vagal tubercle neurons. Copyright © 2018, American Association for the Advancement of Science Publishing Group. Replicated with permission from Ref. [19].

There are two neuroanatomical pathways that facilitate communication between the gut and brain. The first pathway involves the direct communication between the gut and brain through the vagus nerve (VN) in the spinal cord and the autonomic nervous system (ANS) The second pathway involves bidirectional communication between the gut and brain can communicate in both directions through the ENS in the intestinal tract, ANS and VN in the spinal cord [17]. The integration and monitoring of gut function, as well as the connection between the brain's affective and cognitive regions with peripheral gut processes such as immunostimulation, gut permeability, responses, and gut endocrine signaling, are facilitated by the interplay of immunological and neuroendocrine mediators. Furthermore, the stimulation of the VN has been found to have several beneficial effects on the gut microbiota and exhibit anti-inflammatory properties [17]. The brain plays a significant role in regulating various aspects of the communication network, including the microbiota, intestinal motility, sensory, and secretory functions. Conversely, signals originating from the gut and bacteria also exert an influence on brain function [18]. Imbalances in the gut microflora and associated dysfunction in the MGBA have been implicated in the development of numerous neurodevelopmental, cognitive, and neurodegenerative disorders. These abiotic factors have been identified as contributors to brain defects such as behavioral and neuronal dysfunction, leading to the development of AD and other related mental disorders. Moreover, they are closely associated with inappropriate systemic inflammatory responses. In addition to the neurological system's signaling, there exist various communication pathways between the gut microbiota and the brain, including immunological pathways, inflammation-promoting cytokines, short chain fatty acids (SCFAs), and neurotransmitters (NT) produced by the gut microbiota (Fig. 2).

### 2.2 Immune system pathway

The immune system is regulated by the presence of intestinal microflora, which accounts for over 70% of the total immune capacity. The dysregulation of gut microbiota has a profound impact on human homeostasis and overall health due to its influence on intestinal permeability [20]. An overabundance of unfavorable flora or a decrease in microbial diversity can significantly alter intestinal permeability. Consequently, invasive microorganisms, lipopolysaccharides (LPS), and specific metabolites can breach the colon barrier and enter the bloodstream, triggering the release of various inflammatory cytokines by the immune system. The intestinal mucosa's chronic or persistent inflammatory response can potentially overpower the immune system, leading to a chronic, hyperactive yet inefficient immune response, This phenomenon occurs despite the crucial and highly efficient nature of this immune system strategy during emergency situations [21]. Chronic translocation of microorganisms not only elicits an inflammatory response, but also inflicts and exerts an influence on distant organs. Moreover, the integrity of the blood-brain barrier (BBB) integrity was significantly compromised due to bacterial translocation and the release of proinflammatory cytokines, thereby instigating a cascade of neuroinflammatory reactions [22-24]. Microorganisms originating from the periphery possess the capability to infiltrate the brain or reactivate dormant microorganisms within the brain tissue, thereby inducing detrimental chronic inflammation and facilitating the manifestation of AD pathological characteristics, such as the excessive deposition of Aβ. Consequently, this expedites the progression of AD pathology. Furthermore, neuroinflammation can be triggered by the entry of LPS and microbial metabolites that into the brain [25, 26]. Hence, the dysbiosis of gut microbiota not only detrimentally effects the digestive tract, but also significantly disrupts the equilibrium between microflora and immune cells. Consequently, a reciprocal relationship between the intestinal tract and the brain leads to various neurological modifications. A growing body of research suggests that neuroinflammation plays a pivotal role in the initiation of Aβ deposition, tau protein phosphorylation, and eventual neuronal demise [27, 28].

**Figure 3.**
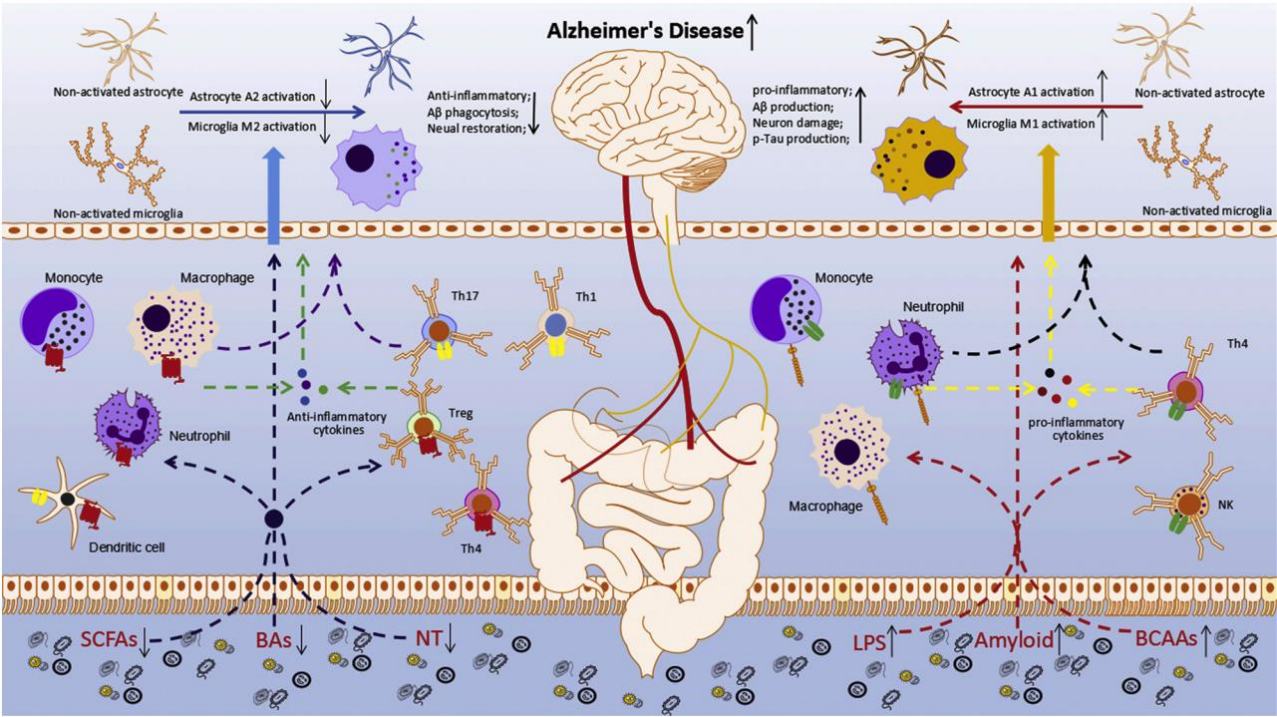
**Gut microbiota accelerates the development of AD through the immune system**. The decrease in anti-inflammatory and increase in pro-inflammatory bacteria in the intestinal flora of AD patients leads to an increase in harmful metabolites and a decrease in beneficial metabolites in the gut. These changes accelerate neuroinflammatory and systemic inflammatory responses in AD patients and reduce Aβ protein clearance and repair of nerve damage, accelerate Tau protein phosphorylation, and Aβ protein deposition, thus accelerating the development of AD. Copyright © 2018, Elsevier Publishing Group. Replicated with permission from Ref. [33].

Over time, pro-inflammatory cytokines, which are initially considered protective agents of the nervous system, undergo a transformation and become implicated as the causative factors of neuropathy[28, 29]. Consequently, the extent of neuropathic damage induced by inflammation is contingent upon the host's degree of activation, stage of pathology, environmental factors, and other variables. SCFAs, which are produced by intestinal microorganisms, during digestion, have the capacity to modulate microglial overactivation by crossing the BBB and triggering the release of numerous inflammatory mediators. These excessive inflammatory reactions serve as the catalyst for the initiation of neurodegenerative processes, subsequently perpetuating and intensifying the ongoing neuroinflammatory cycle [30, 31]. Furthermore, in the presence of an imbalance in the intestinal microecology, certain substances discharged by intestinal microorganisms, including interleukin-6 (IL-6), IL-8, and other inflammatory factors produced by specific *Proteus bacteria*, stimulate the activation of triggering receptors expressed on myeloid (TREM) found on macrophages. This leads to an exaggerated pro-inflammatory reaction and subsequent nerve injury through the modulation of the MGBA [32]. The imbalance of gut microbiota is directly associated with intestinal barrier failure and heightened intestinal permeability. It has been postulated that the pathological progression of the disease is influenced by chronic intestinal inflammation in conjunction with risk factors for AD (Fig. 3).

### 2.3 Pathway of small molecule metabolite delivery system

#### 2.3.1 SCFAs

Evidence from numerous studies suggests that the physiology and behavior of the CNS is influenced by SCFAs, which include propionic acid, butyric acid, and succinic acid, are produced by the fermentation of dietary fiber and sugars by gut microbiota and affect the regulation of fat and glucose metabolism in the body. Research has demonstrated a strong correlation between alterations in the concentrations of SCFAs and the abundance of SCFA-producing bacteria within the gastrointestinal tract, and the onset and progression of neurological disorders, notably AD. SCFAs exert their influence on brain function and behavior through diverse molecular mechanisms, including amelioration of cognitive impairment, modulation of AD-associated marker deposition, regulation of BBB permeability, and provision of anti-inflammatory and anti-apoptotic effects.

In relation to cognitive processes, SCFAs have been shown to enhance the expression of proteins associated with synaptic plasticity. Research has indicated a significant association between histone acetylation and neurodegenerative disorders such as AD. Histone acetylation is jointly regulated by histone acetyltransferases (HATS) and histone deacetylases (HDAC), with HDAC inhibiting histone acetylation and inducing chromatin compaction. Consequently, gene promoters experience reduced accessibility for transcription factors, leading to inducing chromatin compaction. Consequently, gene promoters experience reduced accessibility [35, 36]. The utilization of HDAC inhibitors to regulate HDAC in AD appears to be a promising approach for enhancing gene expression and mitigating AD-associated pathology in affected individuals. SCFAs, being a broad-spectrum HDAC inhibitor, hold significant significance in the inhibition of HDAC [37]. In their study, Govindarajan et al. observed an augmentation in the acetylation of H3K14, H4K5 and H4K12 sites within the hippocampus of APPPS1 double transgenic AD model subsequent to enhanced after sodium butyrate administration. Furthermore, the researchers detected an elevation in the expression of genes associated with synaptic plasticity subsequent to sodium butyrate treatment, thereby implying that sodium butyrate may alleviate cognitive impairment in APPPS1 mice by upregulating the expression of synaptic plasticity-related proteins [38]. Furthermore, Barichello et al. discovered that the administration of sodium butyrate resulted in an augmentation of neurotrophic factor (NF) expression, as well as glial cell line-derived NF, to hereby ameliorating memory impairment caused by experimental pneumococcal meningitis. These findings underscore the pivotal role of NFs in safeguarding neurons and enhancing memory function [39]. The potential mechanism by which sodium butyrate enhances cognitive impairment was investigated. Sodium butyrate mitigates radiation-induced cognitive impairment by mitigates inhibiting hippocampal phosphorylation of cAMP response element binding proteins or promoting the expression of brain derived neurotrophic factor (BDNF) [40]. Additionally, it was observed that sodium butyrate enhances synaptic plasticity through long-term potentiation and depotentiation [41]. In microglia, the augmentation of long-term potentiation and synaptic plasticity is facilitated by sodium butyrate through the up-regulation of the phosphoinositide 3 kinase (PI3K)/ protein kinase B (AKT)/cAMP response element binding (CREB)/BDNF signaling pathway [42]. It is noteworthy to mention that acetate also assumes a significant role in AD. Acetyl-CoA, derived from acetate, serves as an acetyl donor to facilitate histone acetylation and modulate the inflammatory response triggered by microglial activation in the brain, thereby mitigating neuroinflammation in individuals affected by AD [43, 44]. Furthermore, SCFAs exert their influence on the pathological effects of Aβ and Tau through diverse mechanisms. Notably, in a study involving 5×FAD rats treated with sodium butyrate, a dose-dependent decrease in brain Aβ levels was observed in the initial phases of the disease, with the extent of reduction correlating with the dosage of sodium butyrate [38]. However, it should be noted that the administration of sodium butyrate through the lateral ventricles did not result in a decrease in Aβ levels during the advanced stages of the illness [45]. This might be connected to both Aβ's qualities and the sodium butyrate treatment's dosage and duration. Aβ is monomeric in the brain during the early stages of the illness, but as the illness worsens, monomeric Aβ spontaneously aggregates into more toxic and challenging to remove Aβ oligomers or Aβ neural fiber tangles [45]. Sun et al. discovered that sodium butyrate has an impact on mitochondrial function and cell proliferation. This effect is mediated through the regulation of the G protein-coupled receptor (GPR) 109A receptor, inhibition of amyloid precursor protein (APP), promotion of neprilysin (NEP) and BDNF gene expression, and reduction of Aβ damage to cells [46]. Similarly, Filippone et al. observed that sodium butyrate inhibits nitric oxide (NO) synthase and cyclooxygenase-2 (COX-2) , thereby mitigating Aβ-induced damage to nerves and the spinal cord [47]. Furthermore, sodium butyrate has been observed to regulate the hyperphosphorylation of Tau and the expression of inflammatory glial fibrinous acidic protein in cDKO mice, in addition to its impact on Aβ[48].

In terms of inflammation, SCFAs can alleviate the inflammatory response and diminish the levels of pro-inflammatory cytokines. In there in vivo experiments, Soliman et al. observed that acetate led to an augmentation in the acetylation of brain acetyl coenzyme A and histones, while concurrently reducing the activation of glial cells induced by LPS and the expression of IL-1β [44]. Liu et al. discovered that the administration of acetate to APP/PS1 mice resulted in a reduction in G protein expression through the activation of GPR41 and inhibition of the ERK/JNK/NF-κB pathway. Additionally, acetate treatment led to decreased levels of COX-2 and IL-1β, thereby mitigating the neuroinflammatory consequences associated with AD [49]. Moreover, in order to examine the influence of SCFAs on microglial activity, Wenzel et al. utilized differentiated HL-60 monocytes and human THP-1 monocytes as surrogate models for human microglia. In the presence of SCFAs, the levels of inflammatory mediators such as tumor necrosis factor-α (TNF-α) and IL-1β were diminished, and the generation of reactive oxygen species (ROS) was suppressed reduced by inhibiting the respiratory chain triggered by N-formyl-methionine-leucyl-phenylalanine [50]. A comparable finding was reached whereby, following a 4-week treatment involving the administration of water infused with a mixture of SCFAs, a significant restoration in the quantity, functionality, and maturation of microglia within the murine brain was observed [51]. Additionally, Li et al. discovered that SCFAs exhibited a partial reversal of hippocampal neuronal inflammation and damage induced by a high fructose diet, accomplished through the repair of intestinal epithelial barrier impairment and the restoration of NOD-like receptor family pyrin domain-containing 6 (NLRP6) inflammatory body dysfunction in mice [52]. Sodium propionate has been observed to enhance cell survival following antibody stimulation through the reduction of nuclear factor levels in the kappa light peptide gene enhancer of B-cell inhibitory factor alpha, as well as decrease levels of nitrite, NO, and COX-2. These findings suggest an additional molecular mechanism by which SCFAs may contribute to the protection against neuronal injury [47].

**Figure 4.**
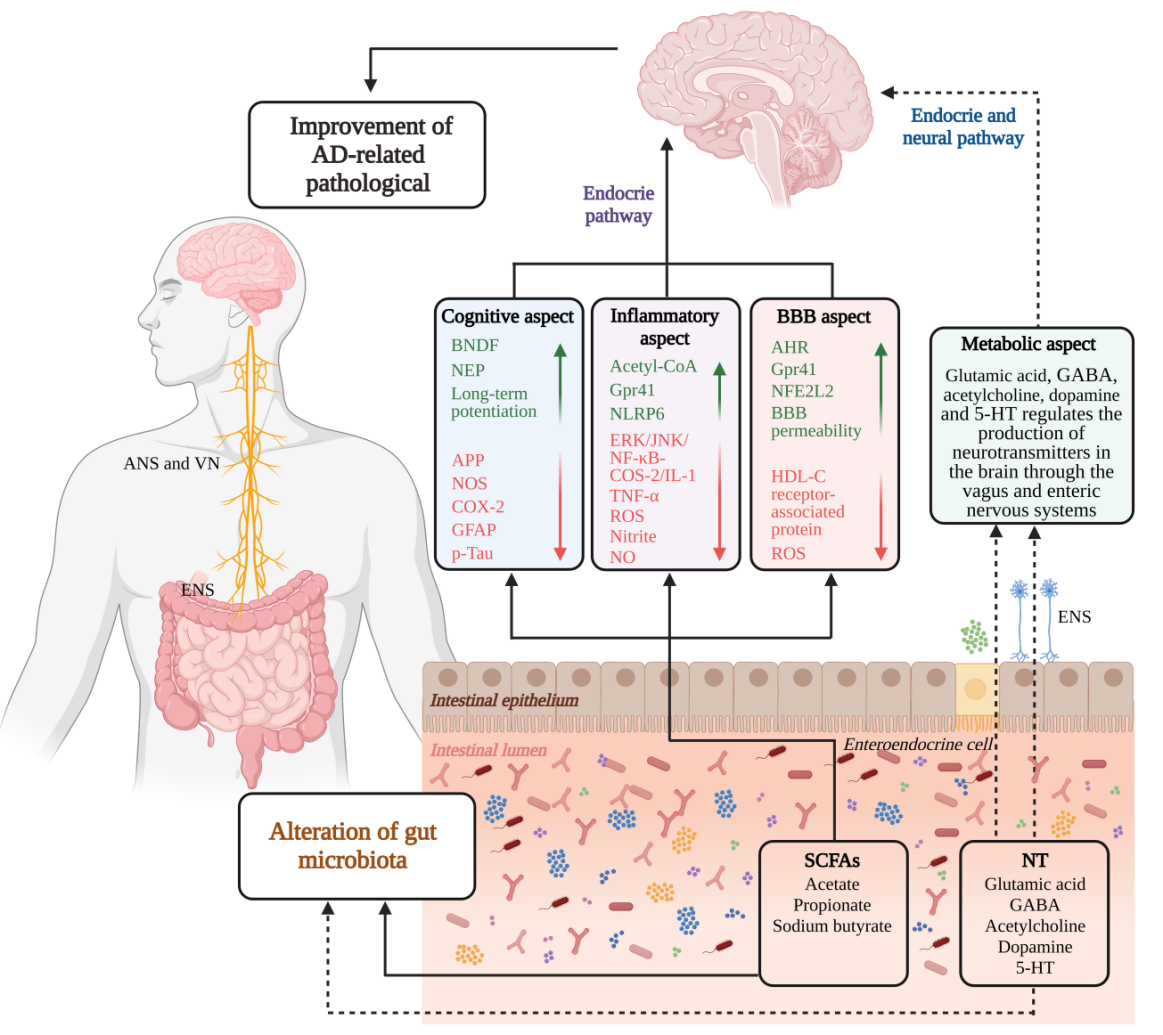
**Gut microbiota accelerates the development of AD through small molecule metabolite delivery system**. In patients with AD, the exchange of information between the intestine and the brain decreases, and due to the decrease of the content of SCFAs and NT produced by the intestine, these beneficial metabolites can not positively stimulate and regulate brain metabolism, accelerating the development of AD. Appropriate supplementation of SCFAs and NT can regulate brain metabolism, slow down the pathological process of AD, and improve cognitive impairment, neuroinflammation and BBB integrity in patients with AD.

In terms of the BBB, SCFAs exhibit a safeguarding and restorative effect on the BBB's functionality and architecture. The presence of sodium butyrate leads to an increased expression of tight junction proteins in the mouse brain. Furthermore, the administration of butyrate-, acetate-, and propionate-producing microbiota to germ-free (GF) mice resulted in a noteworthy enhancement of BBB permeability [53-55]. Furthermore, the aromatic hydrocarbon receptor (AHR), responsible for regulating the metabolic and immunological functions of the gastrointestinal tract, can also be modulated by SCFAs, exhibiting AHR-like properties that mitigate intestinal inflammation and further improve BBB permeability [56, 57]. In addition, exhibits a comparable impact by serving as an activator for GPR41 within the BBB and diminishing the expression of the transporter linked to low-density lipoprotein receptor 1. The oxidative stress (OS) -induced impairment of the BBB is mitigated through the signaling of nuclear factor (erythroid-derived) 2-like 2 [58]. These aforementioned discoveries indicate that the dysregulation of SCFAs resulting from an imbalance in the gut microbiota significantly contributes to the pathophysiology of AD (Fig. 4).

#### 2.3.2 NT

In addition to SCFAs, intestinal bacteria have the capacity to synthesize various NT, including glutamate, γ-aminobutyric acid (GABA), acetylcholine, dopamine and 5-hydroxytryptamine (5-HT) [59-61]. These NT play a crucial role in the neurological and psychological aspects of diverse brain functions, encompassing motor skills, emotional states, cognitive processes, and memory formation [62-64].

Glutamate, an excitatory neurotransmitter, is highly prevalent in the brain and serves a vital function in facilitating the transmission of information among neurons [65, 66]. Research has indicated that, apart from the brain's production of glutamate, certain intestinal endocrine cells within the gastrointestinal tract are also capable of generating glutamate and transmitting stimulation signals to the brain via the VN [19]. This study highlights the role of intestinal endocrine cells in transmitting signals to the VN and promoting the transcription of vesicular glutamate transporter 1 (VGLUT1), leading to the release of glutamate. This rapid transmission of information from the intestine to the brain has been observed. Notably, Frost et al. conducted an experiment involving mice that were administered 13C-labeled inulin orally. The results showed that 13C acetate accumulated in the BBB in the hypothalamus and was involved in the production of glutamate through the neuronal-glial cycle, which is associated with hypothalamic glutamate-glutamine metabolism [67].

GABA plays a crucial role in various physiological processes and brain metabolism, functioning as an inhibitory neurotransmitter that facilitates communication between the gut and brain [68, 69]. Within GABAergic neurons, the enzyme glutamate decarboxylase converts the primary precursor of GABA, glutamate, into GABA [70]. Furthermore, research has revealed that GABA can be synthesized by gut microbiota, stimulating the ENS. In their search for essential growth factors for bacterial life, Strandwitz et al. identified that *Bacteroides fragilis* as a significant producer of GABA, serving as its primary growth factor source [71]. The authors have additionally ascertained that *Bifidobacteria*, *Parabacteria*, and *Eubacteria* can possess the ability to synthesize GABA. It is noteworthy that GABA does not traverse the BBB, and the GABA synthesized by the gut microbiota exerts its effects primarily within the VN or ENS [72]. Importantly, certain metabolites produced by the gut microbiota, such as acetate, can permeate the BBB and accumulate in the hypothalamus, thereby participating in the GABA metabolic cycle [67].

Acetylcholine, a prototypical cholinergic neuro-transmitter, is involved in local mediation role by transmitting excitatory signals between neurons in both the central and peripheral nervous systems in vertebrates [73]. The expression and functioning of acetylcholine are closely associated with neurodegenerative disorders such as AD[74]. Acetylcholine is a commonly occurring metabolite in bacteria, specifically in *Lactobacillus plantarum*, *Bacillus subtilis*, *Escherichia coli*, and *Staphylococcus aureus* [75, 76]. Nevertheless, acetylcholine is unable to traverse the BBB; however, its precursor choline can be transported to the brain through a carrier present on the capillary endothelial cells. Once in the brain, choline can contribute to the biosynthesis of acetylcholine [77].

Dopamine, a catecholamine neurotransmitter, is highly prevalent in the brain and exerts regulatory control over various within the CNS. Dysregulation of the dopamine system significantly contributes to the pathological progression of AD and Parkinson's disease (PD) [78, 79]. Williams et al. found that staphylococcus bacteria residing in the human gut produce substantial quantities of dopamine via the enzymatic activity of aromatic amino acid decarboxylase (SADA) [80]. Further studies by Eisenhofer et al. has demonstrated that the mesenteric organs within the gastrointestinal tract, which are under the influence of the gut microbiota, exhibit substantial dopamine production, constituting approximately 50% of the overall dopamine levels in the body [81]. The disruption of the dopamine system due to alterations in the gut microbiota has the potential to expedite the pathological progression of CNS disorders, including AD and PD.

5-HT, an indole derivative, is predominantly synthesized by intestinal chromophores located in the mucosa of the gastrointestinal tract, constituting 90 percent of the total 5-HT within the body. It serves as a stimulant in the modulation of mood, memory, and overall bodily functions, exerting a positive influence on emotional well-being. Within the intestinal environment, the activity of intestinal chromophores is regulated by the bacterial kynurenine synthesis pathway, facilitating the conversion of tryptophan derived from dietary proteins into 5-HT. Furthermore, it has been observed that bacteria, specifically bacteriophage forming bacteria such as *Clostridium perfringens* in the intestinal tract, can enhance the expression of tryptophan hydroxylase 1 (TPH1), which is the rate-limiting enzyme responsible for the synthesis of 5-HT. This ultimately leads to an accelerated biosynthesis of 5-HT [82, 83]. Luqman et al. have discovered that certain species within the *Staphylococcus* genus possess the capability to decarboxylate the precursor of 5-HT, known as 5-hydroxytryptophan (5-HTP), into 5-HT through the activity of Staphylococcus SADA[84]. Furthermore, the metabolites derived from the microbiota, particularly acetate and butyrate, stimulate the upregulation of TPH1, thereby enhancing the synthesis of 5-HT by intestinal chromaffin cells. Consequently, the and microbial modulation of intestinal homeostasis could be profoundly influenced by the production of 5-HT, , particularly with regards to especially on intestinal motility and platelet function [85-87]. Consequently, disruptions in 5-HT metabolism resulting from imbalances in the intestinal microbiota may expedite the progression of neurodegenerative disorders.

In addition to the aforementioned NT, the brain also harbors a limited quantity of NT, including tyramine and tryptamine among others. Despite their relatively low prevalence in the brain, a significant population of staphylococci capable of synthesizing these trace NT is present in the human intestinal tract [84]. Collectively, these NT play a pivotal role in governing neuronal function and facilitating interneuronal communication within the brain (Fig. 4). In summary, these results demonstrate the irreplaceable role of gut microbiota in regulating host NT and neural signal transduction.

## 3. Treatment strategy of AD based on gut microbiota

The acceleration of the pathophysiological progression of neurodegenerative disorders, such as AD, is facilitated by the gut microbiota. Alterations in the composition and functionality of the intestinal flora have been identified as risk factors for neurodegenerative diseases, both in healthy individuals and patients. Consequently, the investigation of gut microbiota is imperative in order to identify novel therapeutic targets and methodologies for AD. An increasing body of evidence indicates that directing interventions towards the modulation of gut microbiota may present a promising avenue for prevention and treatment of AD. Currently, a diverse range variety of therapeutic interventions has been employed to address gut microbiota disorders, reinstate gut microbiota equilibrium, and enhance the clinical results of diseases such as AD. These interventions encompass the utilization of probiotics, prebiotics and fecal microbiota transplantation (FMT).

### 3.1 Probiotics

**Figure 5.**
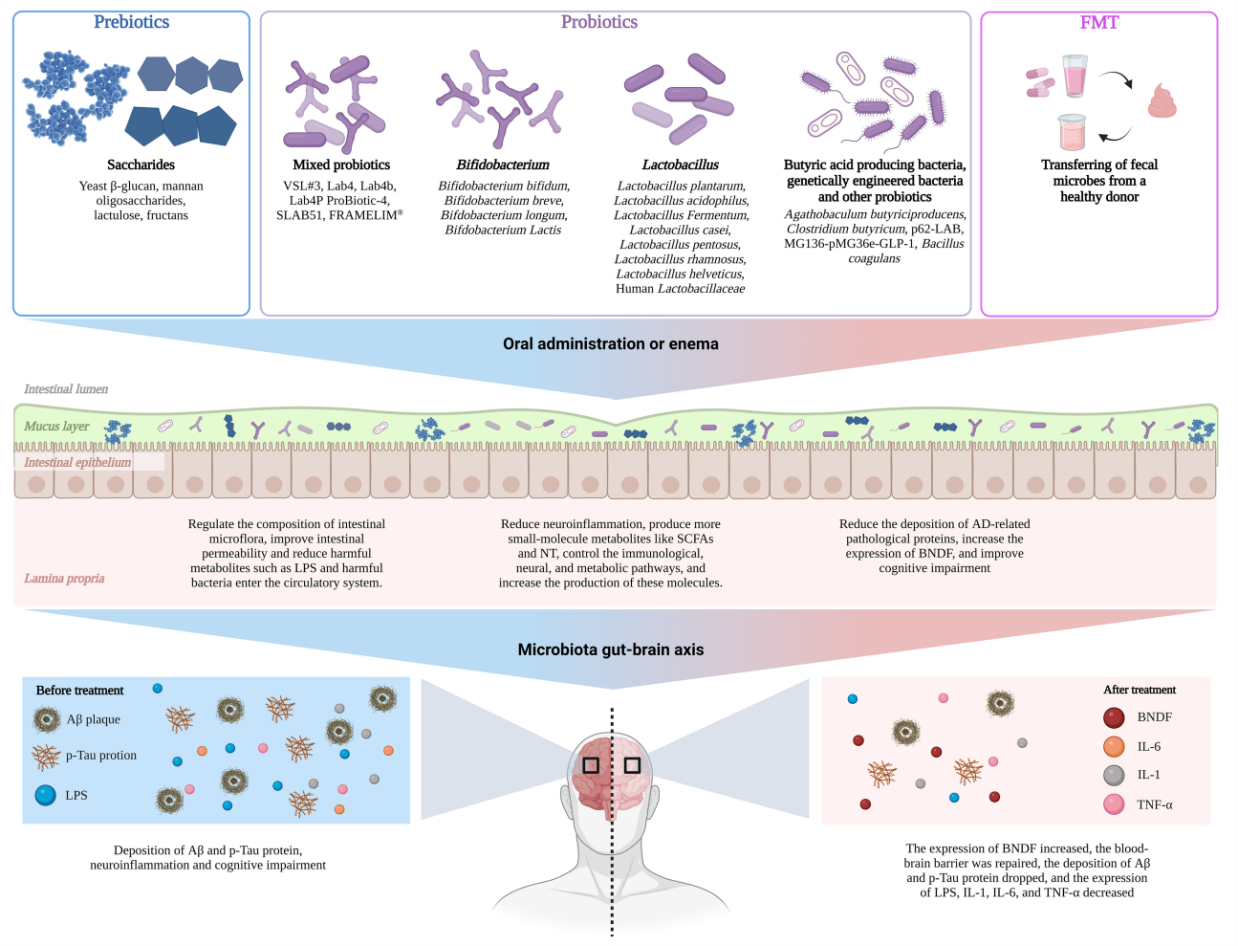
**Improvement effect of probiotics, prebiotics and FMT on patients with AD**. Probiotics, prebiotics and FMT can regulate the composition of intestinal flora, improve intestinal permeability, and increase beneficial metabolites in patients with AD. And reduce neuroinflammation, reduce the deposition of ad-related pathological proteins and improve cognitive impairment in patients with AD through neural, immune, and small molecular delivery systems.

According to the definition published in 1956, probiotics are live microorganisms that exhibit minimal or no pathogenicity and exert beneficial effects on the health of the host[88]. These microorganisms regulate the activity of intestinal cells by modulating specific intracellular signaling pathways, stimulating the production of SCFAs, regulating cell function, enhancing the immune system's defensive capabilities, and reducing the production of inflammatory cytokines. Consequently, probiotics contribute to thereby enhancing the maintenance of gut barrier function integrity and influence the dynamic ecological equilibrium of the gut microbiota (Fig. 5) [89-97]. Additionally, as previously mentioned, the administration of probiotics has been shown to modulate various cognitive, affective, learning, and memory functions within the CNS through the production of SCFAs and NT. Specifically, when the probiotic mixture VSL#3 was administered to animal models with AD, it resulted in an increase in Actinobacteria and Bacteroides within the gut microbiota, a decrease in the expression of markers associated with microglia activation, and an enhancement in the expression of BDNF and synaptic proteins [98]. Moreover, as previously stated, probiotics have the ability to regulate cognitive, emotional, learning and memory processes within the CNS by producing various SCFAs and NTs. In studies involving animal models of AD, the administration of the probiotic mixture VSL#3 resulted in increased levels of *Actinobacteria* and *Bacteroidetes* in the gut microbiota, as well as elevated expression of BDNF and synaptophysin, while reducing indicators of microglia activation indicators [99]. In terms of modulation of intestinal flora metabolites, O'Hagan et al. discovered that the probiotics preparation Lab4, consisting of *Lactobacillus* and *Bifidobacterium*, exerted an influence on the modulation of intestinal flora metabolites. This impact extended to metabolites like GABA and glutamate in the brain, which were altered through multiple pathways. Consequently, the probiotics preparation exhibited the ability to enhance both long-term and short-term memory behavior in rats [100]. The pathological progression of AD is influenced by both mitochondrial dysfunction and an excessive occurrence of OS. A study conducted on transgenic AD mice demonstrated that SLAB51, a combination of Lactobacillus and Bifidobacterium, activated SIRT1-dependent pathways, resulting in a significant reduction of OS in the brain of 3xTg-AD mice [101]. Moreover, SLAB51 exhibited various beneficial effects such as enhancing the production of anti-inflammatory metabolites in the intestine, increasing plasma levels of neuroprotective gut hormones, improving glucose absorption and metabolism, reducing amyloid deposition and brain damage, and ameliorating cognitive impairment [102].

Azm et al. found similar conclusions in their study. They observed a decrease in levels of OS biomarkers and a significant improvement in spatial memory skills in AD mice treated with a combination consisting of *Lactobacillus acidophilus*, *Lactobacillus fermentum*, *Bifidobacterium lactis*, and *Bifidobacterium longum* [103]. *Bifidobacterium* A1 was found to reduce memory behavior and cognitive impairment in AD mice by regulating neuroinflammation and Aβ formation. This was achieved through the inhibition of inflammatory and immunoreactive gene expression in the hippocampus induced by Aβ, either directly by *Bifidobacterium* A1 or its metabolite acetate [104]. Moreover, the *Bifdobacterium* family member, *Bifdobacterium breve MCC1274*, has been found to exhibit a comparable impact on the hippocampus by decreasing the levels of presenilin 1 protein and phosphorylated tau protein [105]. *Bifdobacterium Lactis* Probio-M8 has demonstrated the ability to diminish the accumulation of Aβ plaque throughout the entire brain, mitigate gut microbiota imbalance, and alleviate cognitive impairment in APP/PS1 mice [106]. Additionally, the study revealed that the administration of *Clostridium butyricum* effectively inhibited the synthesis of Aβ and mitigated neuroinflammation. In the context of rats with AD, the introduction of *Clostridium butyricum* successfully prevented cognitive decline, Aβ accumulation, activation of microglia, and the production of TNF-α and IL-1β. Moreover, it demonstrated a reduction in aberrant expression of gut microbiota and levels of butyric acid [107]. *Akkermansia muciniphila* (*A. muciniphila*) exhibited comparable functionalities, as evidenced by its significant amelioration of Aβ protein deposition and intestinal barrier dysfunction in the brains of APP/PS1 mice, and neurobehavioral assessments demonstrated notable enhancements in memory function and mitigation of obesity induced by a high-fat diet [108, 109]. Subsequent investigations unveiled that pasteurized *A. muciniphila* yielded significantly superior outcomes in memory, anxiety, aggression, and social preference compared to untreated *A. muciniphila* [110]. By modulating gut microbiota, the authors also discovered that pasteurized *A. muciniphila* enhanced systemic metabolism and A protein deposition in the brain [110]. SLAB51 exhibited comparable efficacy to *A. muciniphila* in reducing blood glucose and lipids. The administration of SLAB51 resulted in enhanced glucose levels in the brain through the restoration of GLUT3 and GLUT1 expression along with insulin-like growth factor receptor expression. Consequently, this led to dephosphorylation of protein kinase B and adenosine monophosphate-activated protein kinase. At the same time, the treated mice demonstrated a significant decrease of 108% in phosphorylated tau aggregates [111]. As previously stated, the acceleration of the AD pathological process is facilitated by the heightened intestinal permeability caused by dysbiosis of the intestinal flora. In light of this, investigations into the treatment of old and AD mice with probiotics have demonstrated that *Bifidobacterium longum* modifies the composition of the intestinal microbiota, diminishes the presence of LPS in both stool and blood, inhibits the activation of NF-κB, suppresses the expression of TNF-α, and enhances the expression of intestinal tight junction proteins. These findings suggest that *Bifidobacterium longum* mitigates cognitive decline, reduces Aβ protein aggregation and improves intestinal permeability through the modulation of the microbiota [112]. Yang et al. also reached the same conclusion. The researchers observed a significant reduction in intestinal barrier dysfunction and BBB dysfunction in aged mice treated with probiotic-4. Additionally, the levels of IL-6 and TNF-α mRNA and protein were decreased. These effects were specifically observed in SAMP8 animals that received probiotic treatment. Furthermore, the study revealed a decrease in NF-κB translocation, toll-like receptor 4 (TLR4) expression, as well as a reduction in decreased plasma and brain LPS concentration[113]. In addition to natural probiotics, genetically engineered bacteria have also played a role in improving and alleviating the pathological process of AD. The AD mice's brain Aβ levels, as well as neuronal oxidative and inflammatory processes, were considerably enhanced by the engineered probiotics *Lactobacillus lactis*, which carries an encoded human p62 protein [114].

Probiotics have been discovered to have therapeutic effects when mixed with medications in addition to their effects when taken alone. Intestinal flora can be recovered using *Lactobacillus plantarum* and AD together. *Lactobacillus plantarum* prevents the synthesis of trimethylamine-N-oxide (TMAO). The digestive tract produces the microbial metabolite TMAO, which has the ability to accelerate the clinical traits of AD mice and speed up the disease progression [115]. In addition to drug combination, probiotics are also used in combination with other beneficial stimuli. In this regard, the combination of probiotics FRAMELIM^®^ and exercise has been shown to regulate intestinal flora, and the deposition of Aβ in the hippocampus of AD mice is decreased, and memory function is significantly improved [116]. Moreover, the synergistic effect of selenium and probiotics supplements has been uncovered, as they effectively ameliorate cognitive impairment associated with AD by addressing metabolic dysfunctions, mitigating inflammation, and reducing OS [117].

In addition to animal models, clinical studies have yielded similar results. In comparison to the placebo group, AD patients treated for 12 weeks with a probiotics mixture containing *Lactobacillus acidophilus*, *Lactobacillus casei*, *Bifidobacterium bifidum*, and *Lactobacillus fermentum* showed improved levels of plasma malondialdehyde, serum C-reactive protein, beta cell function, serum triglyceride levels, and there were significant differences in the Mini-Mental State Exam (MMSE) [118]. *Bifidobacterium breve* A1 exhibited a comparable function. The participants' scores on the Repeatable Battery for the Assessment of Neuropsychological Status significantly improved after a 16-week intervention with Bifidobacterium breve A1 compared to the placebo group. Additionally, the subjects' scores in the domains of immediate memory, visual space, and memory were significantly enhanced [119]. This finding aligns with the conclusion reached by Asaoka et al [120]. The MMSE and categorical verbal fluency test scores were much higher after a 12-week course of treatment with *Bifidobacterium longum* R0175 and *Lactobacillus rhamnosus* HA-114, and AD-related symptoms were dramatically reduced in AD patients [121]. Furthermore, the administration of combined probiotics, namely *Bifidobacterium longum* BORI BGN4 and *Bifidobacterium bifidum*, effectively mitigated the psychological stress in elderly individuals and substantially elevated the expression levels of BDNF. It is worth emphasizing that BDNF plays a pivotal role in safeguarding neurons, enhancing memory, and reducing neuronal degeneration in the elderly population [122]. Additionally, it has been observed that a number of probiotics exhibit advantageous preventive and enhancing effects on individuals who do not suffer from cognitive impairments. Following a continuous 16 weeks administration of *Bifidobacterium longum* BB68S probiotics, the RBANS scores of the subjects, as well as relative abundance of probiotics such as *Lachnospira*, *Bifidobacterium*, *Dorea*, and *Cellulosilyticum* in the gastrointestinal tract, were found to be significantly higher [123]. Besides, the therapeutic effects of AD have been demonstrated. Ton et al. conducted a study which revealed that the consumption of milk fermented with mixed probiotics led to a significant reduction in inflammation and OS biomarkers in AD patients. Additionally, this intervention improved mitochondrial function, DNA damage or repair, and reduced apoptosis [124]. In addition, a meta-analysis demonstrated that individuals who consumed probiotics experienced enhanced cognitive function and a notable decrease in inflammation and OS-related biomarkers when compared to control group [125, 126]. Table 1 summarizes in detail the application and treatment outcomes of various probiotics in different animal models and clinical settings.

In summary, it has become possible to use probiotics to prevent or improve neurodegenerative diseases such as AD. However, some of its mechanisms remain to be explored.

**Table 1:** Application and therapeutic effect of multiple probiotics in different animal models and clinical environments.

Probiotics	Species	Methods of Administration and Disposal	Probiotics dosage	Experimental Cycle	Study Cohott/Sample Size	Results	Ref.
**VSL#3**	*Streptococcus*, *Bifidobacterium*, *Lactobacillus*	Mixed in maple syrup	12.86 billion living bacteria/kg/day	6 weeks	3-months and 20-22-months old Wistar rat (n=5)	Age-related deficits were reduced in Wistarrats treated with VSL#3, activation indicators of microglia were moderately reduced, expression of BDNF and synaptophysin was up, and *Actinobacteria* and *Bacteroides* were increased in the intestinal microbiota.	[98]
	*Streptococcus*, *Bifidobacterium*, *Lactobacillus*	Mixed in MediGel^®^	0.32 × 10^9^ CFU bacteria/25g mice	2 months	6-8-months old female C57BL/6 wild-type and *App^NL-G-F^* mice (n=15)	Both genotypes' serum concentrations of the SCFAs acetate, butyric acid, and lactic acid rose following treatment with VSL#3, and the c-Fos staining in the hippocampus of *App^NL-G-F^* animals rose as well.	[99]
**Lab4**	*Bifidobacterium*, *Lactobacillus*	Sprinkled on standard laboratory food pellets	1 × 10^8^ CFU/capsule per rat	2 months	15-months-old male Lister Hooded rats (N=14/13)	Lab4 improved the changes of metabolites such as GABA and glutamate in rats' brain and improved the long-term and short-term memory behavior of rats.	[100]
**Lab4b**	*Bifidobacterium*, *Lactobacillus*	Dissolved in forage	5 × 10^8^ CFU/mouse/ day	12 weeks	12-weeks old male 3xTg-AD mice (n=10)	The Lab4b group performed better in new object recognition tests, significantly improved the spinal density of hippocampal neurons, reduced brain and systemic inflammatory response, and regulated intestinal flora.	[127]
**Lab4P**	*Bifidobacterium*, *Lactobacillus*	Lyophilized powder	5 × 10^8^ CFU/mouse/day	14 weeks	Male 3xTg-AD and C57BL/6 mice (n=18)	Probiotics had an anti-inflammatory impact, as shown by Lab4P, which significantly reduced the density of spinous process in hippocampal neurons and the disease-related decline in mRNA expression in hippocampal tissue. Lab4P metabolites also had a protective effect on nerve cells.	[128]
**SLAB51**	*Bifidobacterium*, *Lactobacillus*	Dissolved in water	200 billion bacteria/Kg/day	16 weeks	8-weeks old male 3xTg-AD mice (n=32)	SLAB51 significantly reduces OS in the brains of AD mice through activation of SIRT1-dependent mechanisms.	[101]
	*Bifidobacterium*, *Lactobacillus*	Dissolved in water	2 × 10^11^ CFU bacteria/kg/day	2 and 12 months	8-weeks old male 3xTg-AD mouse (n=24)	SLAB51 enhanced cognitive impairment, glucose absorption and metabolism, decreased Aβ build up and brain damage in 3xTg-AD mouse, and elevated plasma levels of neuroprotective intestinal hormones.	[102]
	***Bifidobacterium*, *Lactobacillus***	Dissolved in water	200 billion bacteria/kg/day	16 and 48 weeks	8-weeks old male 3xTg-AD and wild-type B6129SF2 mice (n=24)	SLAB51 boosted glucose absorption in 3xTg-AD mice by improving phosphorylated tau aggregates and memory function in the brain, and by restoring the expression levels of critical glucose transporters (GLUT3, GLUT1) and insulin-like growth factor receptor in the brain.	[111]
***Akkermansia muciniphila***	*Akkermansia*	Dissolved in PBS	200µL 5 × 10^9^ CFU/mL/day	6 months	3-months old APP/PS1 mice (n=6/10)	*A. muciniphila* can effectively reduce the levels of fasting blood glucose and serum diamine oxidase in APP/PS1 mice, reduce the decrease of colonic mucous cells, improve the level of blood lipids and liver steatosis, promote the decrease of A β level in cerebral cortex and improve memory function.	[108]
	*Akkermansia*	Dissolved in PBS and 30 min at 70 °C	10µL 5 × 10^9^ CFU/mL/day	6-7 months	6-7 months old wild-type zebrafish (n=100)	Pasteurized *A. muciniphila* significantly improved and prevented glycemic, body mass index and diabetic indicators in zebrafish with diabetes combined with AD and alleviated AD-related indicators and significantly improved memory, anxiety, aggression and socially preferred behaviors.	[110]
***Bifidobacterium breve* MCC1274**	*Bifidobacterium*	Dissolved in normal saline	1 × 10^9^ CFU/6.25 mg/200 µL saline/mouse/day	4 months	2-month-old C57BL/6J-AD mice (n=20)	*Bifidobacterium breve* MCC1274 alleviates AD-like pathology in WT mice by reducing the level of presenilin1 protein and phosphorylated tau protein, reducing the level of soluble Aβ 42 in hippocampus, alleviating neuroinflammation and improving the level of synaptic protein.	[105]
	*Bifidobacterium*	Dissolved in normal saline	0.2mL 1 × 10^9^ CFU/mouse/day	4 months	6-months old *App^NL-G-F^* mice (n=26)	By raising the levels of a-disintegrin and metalloproteinase 10, *Bifidobacterium breve* MCC1274 can prevent cognitive decline and decrease Aβ deposition in the hippocampus. Additionally, it weakens the activation of microglia and stimulates the ERK/HIF-1 signal pathway, which lowers the mRNA expression of pro-inflammatory cytokines in brain tissue.	[129]
***Bifidobacterium breve* HNXY26M4**	*Bifidobacterium*	Dissolved in skimmed milk	1 × 10^9^ CFU/mouse/day	12weeks	16-weeks old male APPswe/PS1dE9 mice (n=8)	*Bifidobacterium breve* HNXY26M4 improved the function of the intestinal barrier, reduced neuroinflammation and synaptic dysfunction, improved the composition of the intestinal flora, and lessened the cognitive impairment in APP/PS1 mice.	[130]
***Bifidobacterium breve* A1**	*Bifidobacterium*	Lyophilized capsule	2 × 10^10^ CFU/human/day	16 weeks	Mild cognition impairment patient (n=40)	The total score of RBANS in the *Bifidobacterium breve* A1 group was significantly higher than that in the placebo group, especially in the areas of immediate memory, visual space/structure and delayed memory.	[119]
	*Bifidobacterium*	Lyophilized powder containing cornstarch	2 × 10^10^ CFU/human/day	24 weeks	65-88-years old mild cognition impairment patient (n=55/60)	The MMSE's "orientation in time" and "writing" subscales significantly improved for the *Bifidobacterium breve* A1 group, and *Bifidobacterium breve* A1 also prevented the patient's brain from growing in an unfavorable way.	[120]
***Bifdobacterium breve* strain A1, non-viable components of the bacterium or its metabolite acetate**	*Bifidobacterium*	Dissolved in normal saline, 30 min at 60 °C and sonicate treatment	0.2mL 1× 10^9^ CFU/mL/day	6 days	10-week-old male ddY mice (n=11/12)	*Bifdobacterium breve* strain A1 reversed the impairment of alternating behavior in the Y-maze test and the reduction of delay time in the passive avoidance test, and the non-living component of the bacteria or its metabolite acetate partially ameliorated the cognitive decline in AD mice.	[104]
***Bifidobacterium breve* CCFM1025 and *Bifidobacterium breve* JSWX22M4**	*Bifidobacterium*	Dissolved in skimmed milk	6 × 10^8^ CFU/mouse/day	6 weeks	8-weeks old male C57BL/6J mice (n=8)	*Bifidobacterium breve* CCFM1025 and *Bifidobacterium breve* JSWX22M4 treatment significantly improved synaptic plasticity and increased BDNF and fibronectin type III domain-containing protein 5, and postsynaptic density protein 95, thus delaying the pathological development of AD.	[131]
***Bifdobacterium longum***	*Bifidobacterium*	Dissolved in PBS	1 × 10^9^ CFU/mouse/day	4 and 8 weeks	6-months, 18-months old male C57BL/6 and 5×FAD-Tg mice (n=6)	*Bifdobacterium longum* changed the gut microbiota of 5×FAD-Tg and aged mice, decreased the level of LPS in feces and blood, inhibited the activation of NF- kappa B and the expression of TNF-α, increased the expression of tight junction protein in colon, and inhibited the expression of Aβ, β/γ-secretase, caspase-3 and Aβ accumulation in hippocampus.	[112]
***Bifidobacterium longum* BB68S**	*Bifidobacterium*	Freeze-dried powder	1 × 10^11^ CFU/human/day	8 weeks	healthy elder (n=30)	*Bifidobacterium longum* BB68S intervention increased the relative abundance of the beneficial bacteria *Lachnospira*, *Bifidobacterium*, *Dorea*, and *Cellulosilyticum* and decreased the relative abundance of bacteria associated with cognitive impairment such as *Collinsella*, *Tyzzerella* and *Parabacteroides*.	[123]
***Bifidobacterium bifidum* BGN4 and *Bifidobacterium longum* BORI**	*Bifidobacterium*	Dissolved in sterile water	1 × 10^9^ CFU/mouse/day	30 days	3-months old C57BI/6 and 5xFAD mice (n=10)	*Bifidobacterium bifidum* BGN4 and *Bifidobacterium longum* BORI effectively inhibited amyloidosis and apoptotic processes by improving neuroinflammatory responses and BDNF expression and ameliorated cognitive and memory deficits in AD mice.	[132]
	*Bifidobacterium*	Probiotics capsules containing soybean oil	1 × 10^9^ CFU/human/day	12 weeks	63-years old healthy elder (n=31)	The serum BDNF level was significantly higher in the *Bifidobacterium bifidum* BGN4 and *Bifidobacterium longum* BORI group compared to the placebo group, and both the psychological flexibility test and stress score improved.	[122]
***Bifidobacterium bifidum*, *Lactobacillus plantarum* and exercise**	*Bifidobacterium, Lactobacillus*	Dissolved in water	2 × 10^9^ CFU/mouse/day	8 weeks	8-weeks old male Wistar rat (n=5)	The combination of *Bifidobacterium bifidum*, *Lactobacillus plantarum* and exercise significantly improved Aβ plaque deposition and reduced brain cell death in the brains of AD mice.	[133]
**FRAMELIM^®^ and exercise**	*Bifidobacterium, Lactobacillus*	Direct feeding	120 mg/mouse/day	20 weeks	3-months old male APP/PS1 transgenic mice (n=32)	Exercise and FRAMELIM^®^ supplementation significantly reduced the amount of Aβ in the hippocampus of APP/PS1 mice, improved cognitive level, and regulated intestinal flora imbalance.	[116]
***Bifdobacterium Lactis* Probio-M8**	*Bifdobacterium*	Dissolved in normal saline	1× 10^9^ CFU/mL Probio-M8 at the dose of 0.2 mL/10 g body weight	45 days	4-months old APP/PS1 mice (n=11/12)	*Bifdobacterium Lactis* Probio-M8 can reduce the deposition of Aβ plaque in the whole brain, prevent the imbalance of intestinal flora, and alleviate the cognitive impairment of APP/PS1 mice.	[106]
***Bacillus coagulans*** **JA845**	*Bacillus*	Dissolved in normal saline	1 × 10^9^ CFU/mouse/day	10 weeks	Male ICR mice (n=8)	By regulating NRF2/HO-1 and MyD88/TRAF6/NF-κB, *Bacillus coagulans* JA845 pretreatment can prevent cognitive decline, reduce hippocampal neuronal damage, protect neuronal integrity, reduce the deposition of A and excessive phosphorus of tau in the hippocampus of AD model mice, as well as OS and serum inflammatory cytokines.	[134]
***Lactobacillus acidophilus*, *Lactobacillus*** ***Fermentum*, *Bifidobacterium lactis*, and** ***Bifidobacterium longum***	*Lactobacillus, Bifidobacterium*	Dissolved in water	2g(1×10^10^cfu/g)/day	8 weeks	8-weeks old male Wistar rat (n=12)	By changing the flora, probiotics reduce the levels of OS indicators in Male Wistar rats and significantly enhance behaviors like spatial memory.	[103]
***Lactobacillus acidophilus*, *Bifidobacterium bifidum*, *Bifidobacterium longum* and selenium**	*Bifidobacterium*	Dissolved in water	2× 10^9^ CFU/human/day	12 weeks	AD patient (n=26/27)	Patients in the probiotics supplemented selenium group by altered flora had significantly lower insulin, steady-state IR model, LDL and total/HDL-cholesterol, significantly higher total glutathione and quantitative insulin sensitivity assay indices, and total serum antioxidant capacity and glutathione levels were again increased in the probiotics plus selenium group compared to patients in the selenium supplemented group alone. Modulation of OS biomarker levels in Male Wistar rats and significant improvement in behaviors such as spatial memory were observed.	[117]
***Lactobacillus acidophilus*, *Lactobacillus*** ***casei*, *Bifidobacterium bifidum*, and *Lactobacillus fermentum***	*Lactobacillus, Bifidobacterium*	Dissolved in milk	2 × 10^9^ CFU/g/200 mL/human/day	12 weeks	AD patient (n=30)	Plasma malondialdehyde, serum high-sensitivity C-reactive protein, beta-cell function, and serum triglyceride levels were significantly improved in patients treated with multiple probiotics, and there were significant differences in MMSE score results.	[118]
***Lactobacillus plantarum* DP189**	*Lactobacillus*	Dissolved in normal saline	1 × 10^9^ CFU/mouse/day	10 weeks	8-weeks old male and female ICR mice (n=10)	By raising levels of 5-HT, dopamine, and GABA, *Lactobacillus plantarum* DP189 reduces Aβ deposition and neuronal damage, suppresses tau hyperphosphorylation by controlling the PI3K/AKT/GSK-3β pathway, and controls issues with intestinal microbiota.	[135]
***Lactobacillus plantarum* and memantine**	*Lactobacillus*	Dissolved in PBS comprising 15% glycerol	5 × 10^9^ CFU/mL/mouse/day	12 weeks	8-weeks old male C57BL/6J, 6-months old male wild type and PrP-hAβPPswe/PS1^ΔE9^ transgenic mice (n=15/30)	*Lactobacillus plantarum* and memantine treatment can improve cognitive deterioration, reduce the level of β β in the hippocampus, protect the integrity and plasticity of neurons, and inhibit the synthesis of TMAO.	[115]
***Lactobacillus plantarum* MA2**	*Lactobacillus*	Dissolved in normal saline	1 × 10^8^ and 1 × 10^9^ CFU/kg/day	12 weeks	6-weeks old SPF grade Wistar male rats (n=8)	*Lactobacillus plantarum* MA2 can improve the cognitive impairment and anxiety-like behavior of AD rats induced by D-Galactose/AlCl3, reduce neuronal degeneration and A β accumulation in the brain, regulate gut microbiota imbalance, alleviate intestinal mucosal injury through TLR4/MYD88/NLRP3 signal pathway, and inhibit microglial activation and neuroinflammation.	[136]
***Lactobacillus plantarum* NK151 and *Bifidobacterium longum* NK173**	*Lactobacillus, Bifidobacterium*	Supernatant after centrifugation	1 × 10^9^ CFU/mouse/day	5 days	6-weeks old male C57BL/6 mice (n=7)	The behavioral and cognitive damage brought on by *Escherichia coli* K1 can be considerably reduced by *Lactobacillus plantarum* NK151 and *Bifidobacterium longum* NK173, and the amount of neuroinflammatory markers in the hippocampus can also be decreased.	[137]
***Lactobacillus plantarum, Bifidobacterium bifidum* and interval training**		Dissolved in water	1mL 1 × 10^9^ CFU/mouse/day	8 weeks	Male Wistar rat (n=8)	*Lactobacillus plantarum*, *Bifidobacterium bifidum* and interval training groups of mice showed significantly improved hippocampal cell destruction, neuronal degeneration and short-term memory, and significantly higher BDNF mRNA levels.	[138]
***Lactobacillus pentosus* var. *plantarum* C29**	*Lactobacillus*	50 mM sodium bicarbonate buffer containing 1% glucose	1 × 10^10^ CFU/mouse/day	5 weeks	20-weeks old male C57BL/6J mice	*Lactobacillus pentosus* var. *plantarum* C29 ameliorated D-galactose-induced memory impairment, reversed the inhibition of BDNF and doublecortin expression and cAMP response element-binding protein activation, and reduced the inhibition of aging p16 and inflammatory markers p-p65, p-FOXO3a, cyclooxygenase (COX)-2 and inducible NO synthase (iNOS).	[139]
***Lactobacillus*** ***rhamnosus* HA-114 or *Bifidobacterium longum* R0175**	*Lactobacillus, Bifidobacterium*	Probiotics capsule	1 × 10^15^ CFU/human/day	12 weeks	AD patient (n=45)	*Lactobacillus rhamnosus* HA-114 or *Bifidobacterium longum* R0175 significantly improved the MMSE score of patients.	[121]
***Lactobacillus rhamnosus* UBLR-58 and curcumin**	*Lactobacillus*	Supernatant after centrifugation	1 × 10^6^ CFU/mouse/day	10 days	Scopolamine-induced albino female mice (n=6)	as combined with *Lactobacillus rhamnosus* UBLR-58, curcumin considerably boosted the amount of antioxidant enzymes, reduced neuronal damage, and significantly enhanced memory and cognitive skills as compared to the curcumin group alone.	[140]
***Lactobacillus fermentum* LAB9 or *Lactobacillus fermentum* LABPC**	*Lactobacillus*	Dissolved in normal saline	0.2mL 1 × 10^9^ CFU/mouse/day	28 days	2-months old male ICR mice (n=6)	*Lactobacillus fermentum* LAB9 or *Lactobacillus fermentum* LABPC attenuated LPS-induced memory impairment in mice, increased the level of antioxidants and decreased the levels of MDA, AChE and proinflammatory cytokines.	[141]
***Lactobacillus helveticus* R0052 and *Bifidobacterium longum* R0175**	*Lactobacillus, Bifidobacterium*	Dissolved in normal saline or distilled water	1 × 10^9^ CFU/mouse/day	14 days	Male Wistar rat (n=10)	*Lactobacillus helveticus* R0052 and *Bifidobacterium longum* R0175 significantly improved the level of pro-inflammatory cytokines in hippocampus induced by LPS and alleviated the memory damage induced by LPS by up-regulating the expression of BDNF.	[142]
***Agathobaculum butyriciproducens***	*Agathobaculum*	Dissolved in PBS	2 × 10^8^ CFU/mouse/day	10 weeks	8-weeks old Tg-APPswe/PS1dE9 and C57BL/6J mice	Aβ deposition and microglia activation in the parietal cortex and hippocampus of APP/PS1 mice were significantly reduced after treatment with *Agathobaculum butyriciproducens*, and IL-1 and C1QB gene expression levels in the cerebral cortex were decreased while the gene expression levels of downstream signal pathways were up-regulated. Controlling neuroinflammation and the IGF-1 signal in the animal model enhanced cognitive function.	[143]
***Clostridium butyricum* or butyrate**	*Clostridium*	Dissolved in phosphate-buffered saline (PBS)	200µL 1 × 10^9^ CFU/mL/day	4 weeks	6-months old APPswe/PS1dE9 mice (n=10)	Treatment with *Clostridium butyricum* could stop TNF-α, Aβ deposition, microglial activation, and cognitive impairment in the brains of APP/PS1 mice. In BV-2 microglia that had been activated by A, butyrate therapy reduced CD11b and COX-2 levels and prevented the phosphorylation of NF-κB p65.	[107]
**ProBiotic-4**	*Lactobacillus, Bifidobacterium*	Direct feeding	2 × 10^9^ CFU/mouse/day	12 weeks	9-months old male SAMP8 mice (n=12)	ProBiotic-4 significantly improved memory deficits, brain neuronal and synaptic damage, glial activation, and microbiota composition in feces and brain in aged SAMP8 mice, and significantly attenuated aging-related disruption of the intestinal and BBB, reduced IL-6 and TNF-α mRNA and protein levels, and decreased plasma and brain LPS concentrations, TLR4 expression, and brain NF-κB nuclear translocation.	[113]
**Human** ***Lactobacillaceae***	*Lactobacillus*	Lyophilized powder	1 × 10^9^ CFU/mouse/day	12 weeks	APPswe/PS1dE9 mice (n=12)	Human *Lactobacillaceae* significantly reduced the expression of Aβ plaques, tau phosphorylation and neuroinflammation in the hippocampal region of the mouse brain, increased GSH-PX activity in the brain, decreased the expression levels of IL-6 and MDA, as well as increased the abundance of beneficial bacteria in the gut, inhibited pathogenic bacteria and improved cognitive function in mice.	[144]
**p62 (SQSTM1)-engineered lactic acid** **Bacteria (p62-LAB)**	*Lactobacillus*	Genetic engineering treatment and dissolved in pasteurized skimmed milk	1 × 10^9^ CFU live p62-LAB/mouse/day	2 months	8-weeks old 3xTg-AD mice (n=8)	P62-LAB improved the memory function of 3xTg-AD mice, showing the regulation of ubiquitin-proteasome system and autophagy, the decrease of amyloid peptide level, and the decrease of neuronal oxidation and inflammation.	[114]
**MG136-pMG36e-GLP-1**	*Lactobacillus*	Genetic engineering treatment and dissolved in water	1 × 10^9^ CFU/mouse/day	7 days	Male C57BL/6 mice (n=12)	Through TLR4/NFκB and AKT/ glycogen synthase kinase 3β (GSK-3β) signal pathways, MG136-pMG36eGLP-1 could significantly reduce the memory impairment induced by LPS and motor dysfunction induced by LPS. It also decreased the abundance of pathogens Enterococcus and Proteus and increased the abundance of probiotics *A. muciniphila*.	[145]

### 3.2 Prebiotics

According to the International Scientific Association for Probiotics and Prebiotics, prebiotics are defined as food ingredients that are capable of being utilized by the host microbiota and exert positive effects on human health [146]. Prebiotics, on the other hand, have been employed as supplementary treatments for neurological and psychiatric disorders, including depression, PD, and autism, due to their demonstrated ability to substantially enhance the abundance of beneficial bacteria, such as *Bifidobacteria* and *Lactobacilli*, within the gut microbiota (Fig. 5) [147, 148]. A recent study suggests that prebiotics may have a comparable impact on the prevention and treatment of AD. The study on prebiotics therapy in AD mice found that yeast beta-glucan effectively enhanced the abundance of both pro- and anti-inflammatory bacteria in the gut microbiota, elevated the production of SCFAs, and mitigated neuroinflammation and brain IR [149]. Mannan oligosaccharides have been observed to exhibit comparable effects, including the regulation of intestinal microbiota, enhanced synthesis of SCFAs, improved cognitive abilities, memory function and spatial memory, reduced accumulation of Aβ in the cerebral cortex, hippocampus, and amygdala, as well as substantial mitigation of neuroinflammatory responses and notable adjustment of brain redox equilibrium [150]. Moreover, it has been demonstrated that lactulose enhances cognitive function in AD mice via autophagic and anti-inflammatory pathways [151]. In rat and mouse models of AD, oligosaccharides from Morinda officinalis have been shown to have similar efficacy with improved memory and learning functions and reduced Aβ plaque formation, OS, and overall inflammation levels [152, 153]. In human studies, a study of 1837 healthy older persons without signs of neurodegeneration revealed that daily use of fructans dramatically decreased the risk of acquiring AD [154]. The data supporting the use of prebiotics in clinical practice, even if this study was standardized for subject age, sex, race, daily calorie consumption, education, and ApoE genes, nevertheless lacks validity [155].

In conclusion, although probiotics have demonstrated in mice that they may help in the treatment and prevention of AD, clinical trial data and standardized implementation strategies are still lacking.

### 3.3 FMT

The administration of a treated solution of healthy human

feces, known as FMT, is a method employed to restore the gut microbiota and treat illnesses. Currently, FMT has proven effective in treating *Clostridium difficile* infections [156]. However, trials investigating the potential benefits of FMT in neurodegenerative, inflammatory bowel, and metabolic disorders are still in the preclinical stage and lack clinical trial data. Although these preliminary findings are promising, further research is required to draw definitive conclusions (Fig. 5).

Following the introduction of FMT from healthy mice to AD mice, a decline in neurogenesis and expression of BDNF in the adult hippocampus was observed, accompanied by an increase in p21 expression, deposition of Aβ plaques, and the onset of memory impairment. Furthermore, activation of hippocampal microglia and heightened expression of pro-inflammatory cytokines observed in the mice [157, 158]. Sterile APP transgenic mice exhibited significantly lower levels of Aβ protein in their brains compared to non-aseptic APP transgenic mice within the aseptic animal model [159]. Similarly, after treatment of GF mice with SAMR1 from anti-aging mice versus SAMP8 FMT from non-aging mice, mice in the SAMR1-treated group exhibited improved behavior and indices of gut microbial α-diversity and β-diversity [160]. In human FMT trials, GF mice were transplanted with FMT from both healthy volunteers and patients with cognitive impairments, leading to notably inferior performance in object localization and object recognition tests, as well as had significantly reduced levels of GABA, taurine, and valine in their fecal samples compared to the donors [161]. The results of the study on the treatment of AD mice with FMT demonstrate the significance of gut microbiota in the pathogenesis process and progression of neurodegenerative diseases such as AD. The administration of FMT resulted in improvements in Aβ deposition, neurogenic fiber tangles, neuroglial cell reactivity, and cognitive deficits in the brains of AD mice. Additionally, FMT reversed abnormal intestinal macrophage activity and abnormal expression of circulating blood inflammatory monocyte-related genes in the colon [162]. Another study conducted on mice with AD that were treated with FMT observed a reversal in the alterations of gut microbiota and SCFAs, as well as a decrease in COX-2 and CD11b levels. Furthermore, there was a reduction in the accumulation of Aβ and Tau proteins in the brain, an increase in synaptic plasticity, and a reversal of the changes in gut microbiota and SCFAs[163]. Similarly, Dodiya et al. reached a comparable conclusion and identified changes in the functionality of microglia due to FMT [164]. Furthermore, clinical studies have yielded noteworthy findings alongside animal models. Notably, patients diagnosed with AD exhibited a notable amelioration in gut microbiota and a substantial enhancement in SCFAs levels subsequent to undergoing FMT treatment [165]. These outcomes suggest a significant potential for the application of FMT in the therapeutic management of AD.

In conclusion, despite the evidence provided by FMT regarding the significant involvement of gut microbiota in the initiation and progression of neurodegenerative diseases like AD in mice, the current body of clinical experimental data and standardized implementation procedures remains insufficient.

## 4. Conclusion and perspectives

In the context of treating AD, probiotics, prebiotics and FMT exhibit fewer adverse effects, relatively higher safety profiles, and non-addictive properties compared to alternative therapies. These interventions have the potential to enhance composition of intestinal microorganisms, augment the abundance of beneficial microflora, diminish the presence of detrimental bacteria, mitigate intestinal inflammation, and enhance the functionality of the intestinal mucosal barrier. Nevertheless, drug therapy fails to attain this outcome due to its numerous adverse reactions, high addictive potential, and inadequate safety. Conversely, probiotics, probiotics, and FMT can effectively modulate the levels of SCFAs and NT within the intestinal tract. Consequently, these interventions alleviate the cognitive impairment associated with AD and enhance brain pathology through diverse mechanisms.

The augmentation of intestinal microbiota diversity and abundance through the administration of probiotics therapy or supplements has the potential to improve intestinal health, thereby exerting a therapeutic influence on various diseases, such as AD. There are still certain flaws and drawbacks, though: (I) The lack of adequate, high-quality, multicenter clinical research evidence demonstrating the efficacy of probiotics therapy or supplements in the treatment of AD. (II) The optimal type and dosage of probiotics for treating AD remain uncertain, as do the specific symptoms and pathological stages exhibited by different AD patients. Furthermore, the absence of a standardized implementation strategy further complicates the identification of the most suitable probiotic strain or combination for effective treatment of Alzheimer's. (III) Although probiotics therapy and supplements have been shown to potentially benefit individuals with AD, the precise mechanism underlying their efficacy remains uncertain, necessitating further investigation to elucidate this phenomenon. (IV) Patient survival time and survival rate. There is no concrete proof that probiotics therapy or supplements can increase the survival time of patients with AD or lower their mortality, despite the fact that numerous studies have shown that intestinal flora and pathological traits of AD patients are significantly improved after probiotics treatment. (v) It is imperative to prioritize the monitoring of side effects and safety concerns, particularly individuals with AD concurrently experiencing other disorders. The administration of probiotic therapy or supplements may potentially yield diverse adverse effects, including but not limited to diarrhea, abdominal pain, and flatulence. (VI) Acid-base intolerance. Probiotics often develop in the digestive system. In order to effectively survive and carry out their intended functions within the intestinal system, probiotics necessitate a specific degree of tolerance towards both acidic and alkaline conditions. This is imperative as they undergo digestion by stomach acid and bile upon entry into the gastrointestinal tract. Furthermore, prebiotics pose similar challenges as probiotics in terms of their impact on drug absorption, as they have the potential to alter intestinal pH and disrupt intestinal barrier function. Consequently, this can diminish the effectiveness of certain medications or result in unfavorable reactions. Hence, it is imperative to undertake meticulous multicenter clinical research, investigate the precise mechanisms underlying probiotics and prebiotics, and consider long-term biocompatibility. Moreover, the utilization of appropriate technical approaches or drug delivery systems is necessary to enhance the bioavailability and efficacy of prebiotics. To enhance the acid and alkali resistance of prebiotics supplements, researchers employed various technical approaches, including microencapsulation and coating to envelop probiotics within a protective layer. This technique effectively augments the strain's survival rate in gastric acid and bile, thereby enhancing its bioavailability and efficacy. Nevertheless, the utilization of these methods in prebiotics therapy for patients with AD remains scarcely documented.

Intestinal illnesses are treated using FMT, which has been the subject of extensive research. FMT has drawn attention in recent years as a potential treatment for AD, although there is currently little research being done in this area and many flaws and shortcomings. The selection of donors and the composition of intestinal flora of donors are very important to the effect of FMT. The diversity and individual variations of gut flora, however, it is not easy to select a suitable donor, which may affect the efficacy of FMT. The safety concerns associated with FMT encompass infectious diseases and immune-related reactions, patients with AD commonly present with comorbidities and diverse pharmacological interventions, which can potentially give rise to severe adverse reactions. Consequently, a more comprehensive assessment and surveillance of FMT's safety profile is imperative. The absence of established protocols for FMT poses challenges in replicating findings from previous studies and comparing results across different clinical investigations. The lack of a standardized FMT methodology for AD may lead to variations in procedures and dosages employed, potentially influencing the efficacy and safety of FMT interventions. FMT also involves the topic of allogeneic and xenogeneic transplantation. Allogeneic transplantation can lead to intestinal immune rejection, graft failure, or adverse reactions due to inter-individual variations in gut microbial communities. Consequently, allogeneic transplantation exhibits a superior success rate compared to xenogeneic transplantation.

To sum up, probiotics, probiotics and FMT are relatively safe, effective, with few side effects, and can regulate systemic and brain metabolism from bottom to top to improve the pathology of AD. Probiotics and FMT are both utilized in varying degrees, with probiotics being more prevalent. Furthermore, the therapeutic efficacy of probiotics appears to be superior. Considering the potential involvement of intestinal flora in AD progression and its potential influence on AD treatment, forthcoming research endeavors will ascertain the modulation of gut microbiota and its integration with other therapeutic interventions for AD patients, aiming to enhance the overall therapeutic outcome.
